# Determining influence, interaction and causality of contrast and sequence effects in objective structured clinical exams

**DOI:** 10.1111/medu.14713

**Published:** 2022-01-11

**Authors:** Peter Yeates, Alice Moult, Natalie Cope, Gareth McCray, Richard Fuller, Robert McKinley

**Affiliations:** ^1^ School of Medicine Keele University Keele UK; ^2^ Fairfield General Hospital Pennine Acute Hospitals NHS Trust Bury UK; ^3^ School of Medicine University of Liverpool Liverpool UK

## Abstract

**Introduction:**

Differential rater function over time (DRIFT) and contrast effects (examiners' scores biased away from the standard of preceding performances) both challenge the fairness of scoring in objective structured clinical exams (OSCEs). This is important as, under some circumstances, these effects could alter whether some candidates pass or fail assessments. Benefitting from experimental control, this study investigated the causality, operation and interaction of both effects simultaneously for the first time in an OSCE setting.

**Methods:**

We used secondary analysis of data from an OSCE in which examiners scored embedded videos of student performances interspersed between live students. Embedded video position varied between examiners (early vs. late) whilst the standard of preceding performances naturally varied (previous high or low). We examined linear relationships suggestive of DRIFT and contrast effects in all within‐OSCE data before comparing the influence and interaction of ‘early’ versus ‘late’ and ‘previous high’ versus ‘previous low’ conditions on embedded video scores.

**Results:**

Linear relationships data did not support the presence of DRIFT or contrast effects. Embedded videos were scored higher early (19.9 [19.4–20.5]) versus late (18.6 [18.1–19.1], *p* < 0.001), but scores did not differ between previous high and previous low conditions. The interaction term was non‐significant.

**Conclusions:**

In this instance, the small DRIFT effect we observed on embedded videos can be causally attributed to examiner behaviour. Contrast effects appear less ubiquitous than some prior research suggests. Possible mediators of these finding include the following: OSCE context, detail of task specification, examiners' cognitive load and the distribution of learners' ability. As the operation of these effects appears to vary across contexts, further research is needed to determine the prevalence and mechanisms of contrast and DRIFT effects, so that assessments may be designed in ways that are likely to avoid their occurrence. Quality assurance should monitor for these contextually variable effects in order to ensure OSCE equivalence.

## Abstract 2

Examiner DRIFT and contrast effects are well established effects in #AssessorCognition but how pronounced are they in real #OSCEs? Yeates et al found muted evidence and discuss why effects may vary between settings.

## 1 INTRODUCTION

Ensuring that assessment scores fairly represent the performance of trainees remains a priority for assessment in health professionals' education. Although different philosophical^1^ and epistemological^2^ positions can be adopted to account for variability in assessors' judgements,^3^ the field of assessor cognition has demonstrated influences which can contribute unhelpful variability or bias to assessment judgements, regardless of adopted philosophical stance.^4^ The influences ‘differential rater function over time (DRIFT)’^5^ and ‘contrast effects’^6^ are difficult to ascribe to the notion of ‘meaningful difference’ in experts' judgement^7^ and consequently represent detrimental influences on assessors' judgements. Despite the potential implications for candidates and trainees of these effects, they remain incompletely understood. The purpose of this paper is to extend that understanding and explore it within the context of an objective structured clinical exam (OSCE).

Consideration of these effects should occur in light of what is already known about sources of variance in OSCEs. Variance due to stations is typically the largest systemic source of variance, accounting for approximately 3.5 times the variance due to resident ability (46.7% vs. 13.3% respectively^8^) in one study. Variance due to examiners and simulated patients are often nested in (i.e., confounded with) station variance, making them hard to estimate,^9^ but available estimates of examiner variance vary substantially across studies from trivial (0.4%^10^) to more substantial (13%^11^ or 18%^12^). Notably, contrast effects could erroneously contribute to candidate variance estimates, although DRIFT effects would be expected to contribute to the error term. As a result, neither is routinely demonstrated by conventional psychometric analyses.

Contrast effects describe examiners' tendency to be biased away from the standard of preceding performances, that is, to allocate unduly low scores for one candidate following a good performance of another and unduly high scores following a poor performance.^6^ The effect has been demonstrated in three separate experimental studies, all situated within a workplace‐based assessment context.^6^, ^13^, ^14^ In these studies, contrast effects typically showed a moderate effect size (Cohen's *d* = 0.6) and accounted for a greater proportion of score variance (24%) than examiners' consistent tendency to give either high or low scores (18%; i.e., their ‘Hawkishness’ or ‘Dovishness’), whilst accounting for a 31% difference in pass/fail decisions for borderline candidates.^6^ Further work demonstrated that assessors' narrative judgements were as equally susceptible to the effect as their scores,^14^ whilst other work suggested the effect is likely to operate unconsciously, beyond examiners' awareness.^13^ Although these studies were all experimental and focused on an assessment context of Mini‐CEX assessments of consultation skills, a further study used observational methods to examine patterns of data from an OSCE and a multiple mini‐interview (MMI) for selection.^15^ It found patterns of correlations in both contexts that were consistent with contrast effects, albeit explaining a smaller proportion of variance of between 5% and 11% of score variance across contexts.

As a result, contrast effects appear to be a robust phenomenon with potential to bias examiners' judgements to a small or moderate extent in any setting where trainees are examined in sequence and significantly alter outcomes for candidates near to the pass/fail threshold. Despite this, little further research has explored their impact on practice or attempted to mitigate their effect, particularly in high stakes performance assessments such as the OSCE.

DRIFT is described as a tendency for raters to systematically alter their scoring for progressive candidates over the course of a period of examining.^5^ Mclaughlin et al.^5^ showed that examiners became progressively more lenient across a formative 10 station OSCE, with scores increasing by an average of 0.88% per station. Although this effect appears small at a station level, it meant that residents scored an average of 8.8% higher if they took a station at the end of the OSCE than the start. Having discounted warm up effects, by replicating findings after excluding initial stations, the authors suggested the effect was due to examiner fatigue. By contrast, Hope and Cameron^16^ showed the opposite effect: Examiners in a broadly focused summative undergraduate OSCE (Year 3 of 5) grew progressively more *stringent* over time, with a decline of 0.14% per station. This accounted for a 3.27% reduction in scores between the first and last groups. Cotzee and Monteiro^17^ examined these patterns in a summative OSCE that determined whether international nursing graduates could practice in Ontario, Canada. Although they found no general support for DRIFT in these data, they demonstrated a significant negative relationship for one out of 12 stations, which itself appeared to be attributable to one track (and potentially therefore one examiner). Candidates in this track more frequently failed the station when examined late rather than early in the sequence. DRIFT effects therefore can be an important influence on both outcomes and scores but are unpredictable, varying both in direction and occurrence between settings.

Importantly all three studies investigating DRIFT effects used observational data with the authors presuming the observed effects were due to changes in *examiners'* behaviour. Without experimental control, they could not exclude the possibility that the observed effects were due to changes in either *students'* behaviour or other unknown factors. For instance, rather than examiners becoming more lenient with time,^6^ students' performance may have improved over the course of the OSCE. Consequently, it would be useful to determine whether the observed effects are indeed due to examiners.

In summary, contrast effects have predominantly been studied in an experimental context with less insight into their operation in practice and some suggestion that the effects in practice may be smaller. Conversely, DRIFT effects have only been demonstrated observationally without the ability to causally attribute them to examiner behaviour. The aim of this study was to study both phenomena simultaneously within the same OSCE to determine the magnitude of both effects, whether they interact and whether DRIFT effects can be causally attributed to examiner behaviour.

## 2 METHODS

### 2.1 Assessment context

We used secondary data analysis to address this aim, using data from a recent study by Yeates et al.^18^ derived from a summative Year 3 undergraduate OSCE exam at Keele University Medical School. Students were studying for the qualification MBChB, which is a 5‐year, predominantly undergraduate, course. Year 3 is the first year that students spend predominantly in clinical placements and have learned clinical skills appropriate to a broad range of medical, surgical and primary care disciplines. Students had a median age of 22 years (range 20–32 years). The OSCE consisted of 12 × 10 min stations, each student doing four stations on 3 consecutive days. One hundred and thirteen students were examined, distributed across four parallel circuits that were repeated in the morning and afternoon with (predominantly) different examiners. This gave eight separate groups of examiners. Scores were allocated using Keele's GeCos marking system^19^ that collects ratings on five domains (scored 1–4) and a global grade (1–7) on each station, giving a possible score range on each station from 6 to 27. As a consequence of these design features, the OSCE context was somewhat different to the workplace‐based assessment in which the majority of observations of contrast effects have previously occurred^6^, ^13^, ^14^: Examiners used domain‐based ratings with task‐specific prompts rather than generic marking scales; examiners were supplied the correct diagnosis for the case rather than having to reach their own diagnosis and were briefed on the scoring format and had previously (several months earlier) undergone generic benchmarking‐based training, which involved scoring videos of OSCE performances within a faculty development event and comparing and discussing scores.

### 2.2 Dataset

Yeates et al.'s study addressed a different aim to the present study, namely, to compare and adjust for examiner differences across different circuits in a multi‐circuit OSCE exam. Videos of student performances were obtained for each station by filming a small volunteer cohort of students in the morning. Examiners scored videos of student performances in addition to usual scoring of live candidates. The authors used these video scores within statistical analyses to compare and adjust for examiner effects.

**FIGURE 1:**
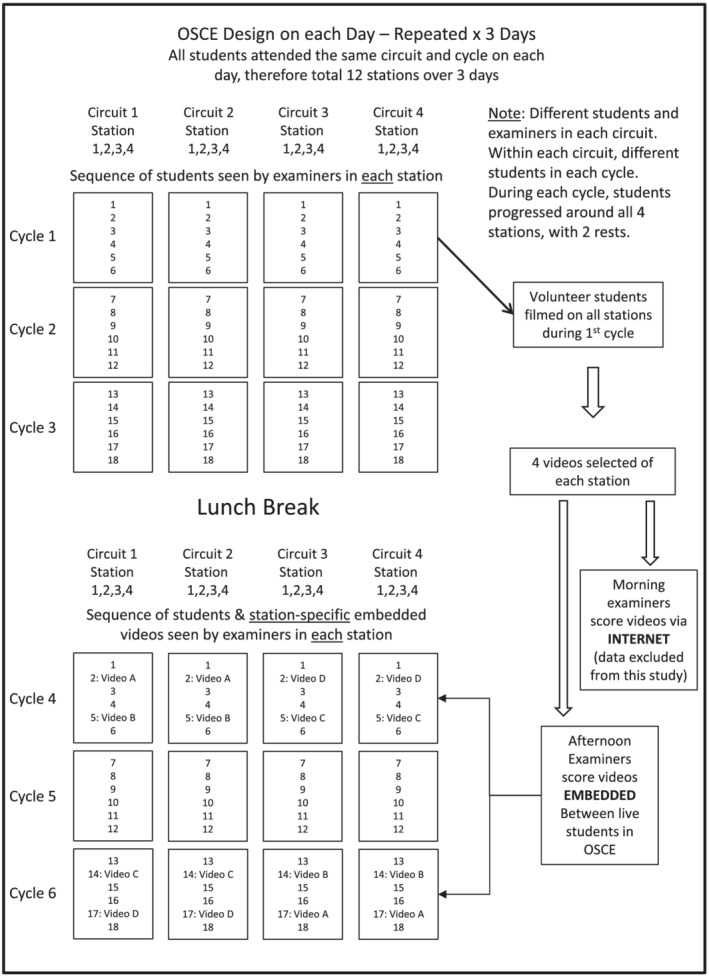
Schematic illustrating the sequence of students seen by each examiner and the position of embedded videos

Half the examiners in Yeates et al.'s study viewed these videos on tablet computers interleaved between live candidates (embedded videos), whilst the other half of the examiners viewed the videos later via the internet after the OSCE was complete. Moreover, although the videos for each station were the same for all groups of examiners, the position of the embedded videos within the OSCE sequence varied for different groups of examiners with some viewing a particular video early in the sequence whilst other examiners viewed the same video late in the sequence of performances (i.e., half of participating examiners scored videos A&B early in the sequence and videos C&D late in the sequence whilst the other half scored videos C&D early in the sequence and videos A&B early in the sequence). Consequently, as Yeates et al.'s^18^ comparisons were derived from the combined scores allocated to both early and late videos, the balanced nature of this variation in embedded video sequence would not be expected to have influenced their comparisons. Nonetheless, this variation in embedded video sequence position enables comparison of scores allocated to the same performance when scored either early or late in the assessment sequence. Additionally, as each video was preceded by a number of live performances, natural variability in these performances meant that in some instances, a video was preceded by comparatively strong performances although in other instances, a video was preceded by comparatively weak performances. This enabled us to determine the presence of both DRIFT and contrast effects in these data with the benefit of experimental control. See Figure 1 for a schematic diagram illustrating the sequence of students seen by examiners and the positions of embedded videos.

Yeates et al. reported no systematic difference between live and video performances for a subset of examiners who scored the same students in both formats (i.e., they scored the videoed students live during the OSCE and then later re‐scored the same performances via video, in order to compare video vs. live scoring). Examiner participation was voluntary, and 76% of examiners took part in the original study. Scores allocated to videos comprised 17.7% of the total data.

### 2.3 Analysis

Using these data, we first attempted to replicate the patterns of observational relationships shown in prior work, which are consistent with contrast and DRIFT effect. As well as aiming to replicate prior work, these analyses made use of all ‘live’ OSCE scores and the scores given to all embedded videos, (i.e., all scores allocated by examiners during the OSCE, hereafter referred to as ‘all within‐OSCE data’) so might be expected to be maximally powered. Second, we examined scoring patterns for the embedded videos to determine whether they showed evidence of contrast or DRIFT effects or an interaction between the two effects.

To examine linear relationships suggestive of contrast or DRIFT effects in the entire dataset, we organised all data collected during the OSCE (all live scores and embedded video scores) in terms of the sequence of performances seen by each examiner. This created a new variable for each performance which we termed ‘sequence’. This ranged from 1 (the first performance seen by a particular examiner) to up to a maximum of 17, the last performance seen by that examiner within a given session of the exam. The maximum sequence value varied between examiners depending on the arrangements of candidates within the session and whether the examiner opted to score embedded videos, between a maximum of 12 and 17. To operationalise contrast effects, based on the methodology in Yeates et al.,^15^ we calculated the average score given to the three preceding candidates by each examiner. This gave a new continuous variable which we termed ‘previous candidates’. We used this measure rather than simply the score of the single previous performance as it consistently showed stronger relationships in Yeates et al.'s^15^ study. Where less than three performances were available, again as per the method of Yeates et al., we used the average of all preceding performances (i.e., the first performance was excluded; for the second performance in the sequence, we used the score for the first performance; for the third performance in the sequence, we used the average of the first and second performances in the sequence). To avoid results being confounded by Simpson's paradox whereby unnecessary causation might be attributed to a single set of data,^20^ we modelled the influence of multiple known predictors of OSCE scores: candidate, station and examiner within a generalised linear model (GLM).^21^ GLM is a statistical method that determines the influence of a number of predictor variables on a dependent variable and that has the flexibility to model random, hierarchical and repeated measures variables as either continuous or categorical predictors (for a general summary, please see Field, Chapter 19^21^). The dependant variable was the score for each performance (continuous), and predictor variables were candidate (nominal), station (nominal), examiner (nominal), previous candidates (continuous) and sequence (continuous). We did not model any interactions. Analysis used maximum likelihood estimation and was performed in IBM SPSS v 26.^22^ These analyses tested two specific hypotheses:

Hypothesis 1 Overall data will show a negative linear relationship between scores and position in the sequence of performances.

**TABLE 1:** Parameter estimates for categorical and continuous variables in generalised linear regression model

	Min value (95% CIs)	Max value (95% CIs)	Wald *χ* ^2^	df	p
Candidate	14.3 (11.9–16.8)	23.0 (20.6–25.4)	409.2	111	0.00
Station	13.6 (11.7–15.5)	25.0 (21.9–28.2)	58.5	8	0.00
Examiner	12.3 (8.6–16.0)	26.9 (22.8–31.0)	259.7	77	0.00

	*β* coefficient	Standard error	Wald *χ* ^2^	*df*	*p*
Sequence	−0.06	0.03	2.8	1	0.09
Previous candidates	−0.02	0.04	0.1	1	0.71
Intercept	25.31	1.70	524.7	1	0.00

Abbreviation: CI, confidence interval.

By way of illustration, Hypothesis 1 would hold true if examiners within a given session of the OSCE became progressively more stringent over time.

Hypothesis 2 Overall data will show a negative linear relationship between scores and the average standard of preceding performances.

To further illustrate, Hypothesis 2 would hold true if examiners were influenced by contrast effects (i.e., their scores were biased away from the standard of preceding performances.

To examine the influence of contrast and DRIFT effects on the scores that examiners gave to the embedded videos, we developed two categorical variables related to sequence and previous candidates. For simplicity, we labelled these variables ‘sequence’ and ‘contrast’. As the median sequence value in the overall data was 8, we denoted performances with low sequence values (1–8) as ‘early” and later sequence values (>8) as ‘late’ to give the sequence variable.

To develop the ‘contrast’ variable, we categorised scores for each embedded video based on the average scores given to the (up to) three preceding candidates. To do this, we compared the value of the ‘previous candidates’ variable with the average score given to the embedded video in question by all examiners (i.e., our best measure of the standard of the embedded video). Instances where an examiner scored an embedded video that had been preceded by comparatively weak performances were denoted ‘previous low’, whereas instances where an examiner scored an embedded video that had been preceded by comparatively strong performances were denoted ‘previous high’. This categorised embedded video scores relative to the standard of the preceding performances regardless of their absolute level (i.e., the score given to an embedded video were categorised as ‘previous high’ if the preceding performances had been scored more highly than it, regardless of whether these performances were actually ‘good’ or not). This relative approach can be justified as Yeates et al.^13^ showed that contrast effects operate at multiple levels of performance, rather than just for borderline performances. Instances where the preceding performances received the same average score as an embedded video were omitted.

Having categorised data, we used GLM to examine the influence of performance (factor), examiner (factor), sequence (factor, early or late) and contrast (factor, previous high or previous low) on the dependent variable of score for embedded video performances. In our first analysis, all factors were compared as main effects without interactions. We then repeated the analysis, including an interaction of Sequence (factor, early or late) × contrast (factor, previous high or previous low). Both models were estimated using maximum likelihood estimation in IBM SPSS v 26.^22^ These analyses tested the following hypotheses:

Hypothesis 3 Examiners will allocate higher scores to embedded videos in the ‘early’ sequence variable condition than in the ‘late’ sequence variable condition.

In practical terms, Hypothesis 3 would hold true if examiners scored a given performance more highly when encountered early in the sequence compared with encountering the same performance later in the sequence.

Hypothesis 4 Examiners will allocate higher scores to embedded videos in the ‘previous low’ contrast variable condition than in the ‘previous high’ contrast variable condition.

In practical terms, Hypothesis 4 would hold true if examiners scored a given performance more highly when it was preceded by comparatively weak performances and lower when it was preceded by comparatively strong performances.

The interaction of Hypotheses 3 and 4 could be hypothecated in either direction on theoretical grounds due to either ‘warm up’ effects (greater evidence of contrast effects early in the sequence) or examiner fatigue (greater influence of contrast effects late in the sequence). Arbitrarily we hypothecated that:

Hypothesis 5 The difference between scores allocated by examiners under the ‘previous high’ and ‘previous low’ conditions will be greater for the ‘late’ condition than the ‘early’ condition.

For inferential statistical tests, we adopted a type 1 error rate of 5% but applied the Bonferroni correction to account for our five separate hypotheses resulting in a significant level of *p* = 0.05/5 = 0.01. We opted not to perform a post hoc calculation of the apparent power of the study as these are sample dependent and therefore have the potential to mislead.^23^ We could have modelled the power of a sample of this size to detect an arbitrary pre‐specified difference; however, owing to the complex data structure, this would have required simulation that would have relied on multiple assumptions (likely derived from sample‐dependent estimates). For these reasons, consistent with Colegrave and Ruxton and Levine and Ensom.,^24^, ^25^ we assert that the 95% confidence intervals (CIs) of the estimates are the best measure of the precision of the analysis and have reported those.

## 3 RESULTS

Included data for the variable ‘sequence’ ranged from 2 to 17, with a uniform distribution, a median of 9 and an interquartile range (IQR) of 8. Data for sequence 1 scores were omitted as they never had corresponding ‘previous candidate’ data. ‘Previous candidates’ data (i.e., the average of the up to three preceding candidates) ranged from 11.3 to 27.0 and was normally distributed (mean–median = 0.09 scale points [0.44%], skewness = 0.11), with a mean of 19.4 and a standard deviation of 2.8. The dependent variable ‘score’ ranged from 7 to 27 and was normally distributed (mean–median = 0.51 scale points [2.4%], skewness = −0.09), with a mean of 19.5 and a standard deviation of 3.7. The maximum scale value of 27 had a cumulative probability function of 0.979 within this distribution, suggesting that 2.1% of observations in the normal distribution would have been expected to exceed this maximum value, suggesting a trivial ceiling effect. The score distribution showed a kurtosis value of −0.26, indicating that there was no significant impact of range restriction on the data, in comparison with a normal distribution. We separately plotted the dependent variable (score) against both continuous predictor variables (1/sequence and 2/previous candidates) to check for evidence of curvilinearity. Although curvilinear relationship may only be apparent in very large datasets, no curvilinearity was apparent and a linear model appeared the most parsimonious solution.

### 3.1 Regression analysis using all within‐OSCE data

GLM results showed that the anticipated categorical predictors (candidate, station and examiner all significantly influenced scores. Scores by candidate ranged from 14.3 (95% CIs 11.9–16.8) to 23.0 (20.6–25.4), Wald *χ* ^2^ 409.2 (*df* = 111), *p* < 0.001. Scores by station ranged from 13.6 (11.7–15.5) to 25.0 (21.9–28.2), Wald *χ* ^2^ 58.5 (8), *p* < 0.001. Scores by examiner ranged from 12.3 (8.6–16.0) to 26.9 (22.8–31.0), Wald *χ* ^2^ 259.7 (77), *p* < 0.001. The variable ‘sequence’ (denoting examiner DRIFT effects) was non‐significant: *β* coefficient = −0.06, *SE* = 0.03, Wald *χ* ^2^ 2.8 (1), *p* = 0.09. As a result, Hypothesis 1 was not supported, and this analysis was not consistent with the presence of DRIFT effects in these data. The variable ‘previous candidates’ (denoting contrast effects) was non‐significant: *β* = −0.016, *SE* = 0.04, Wald *χ* ^2^ 0.2 (1), *p* = 0.71. As a result, Hypothesis 2 was not supported, and this analysis was not consistent with the existence of contrast effects in these data. Please see Table 1 for regression coefficients.

### 3.2 Factorial comparisons of embedded videos scores

Due to voluntary examiner participation in the original study, scores were available for 157 out of a potential maximum of 192 (82%) embedded video performances. Data was provided by 38 unique examiners out of a potential maximum of 48 (79%). Embedded videos were viewed by participating examiners between Positions 1 and 8 in the sequence (i.e., ‘early’) on 68 (43.0%) of occasions and greater than Position 8 in the sequence (i.e., ‘late’) on 89 (57%) of occasions. The imbalance in group sizes between the early and late groups occurred due to a technical failure that resulted in performances being shown to some examiners later in the sequence then intended. Embedded video performances for participating examiners were preceded by comparatively weaker performances (i.e., ‘previous low’) on 83 (53%) occasions and comparatively stronger performances (i.e., ‘previous high’) on 74 (47%) occasions. The imbalance in group sizes in the ‘previous high’ and ’previous low’ groups is expected to have arisen due to natural variations in the performances of preceding students for the subset of examiners who chose to participate. Supporting the intended construct, preceding performances were, on average, 3.6 points (13%) below the average score of the relevant embedded video performance (range 0.1–14 points, *SD* = 2.7) in the previous low group whereas preceding performances were, on average, 3.1 points (11%) above the average score of the relevant embedded video performance (range 0.1–11 points, *SD* = 2.1) in the previous high group. Investigating these data further, the average standard of the three preceding performances was below the pass/fail boundary for the relevant station on 30 out of 83 (36%) of occasions in the ‘previous low’ performances, whereas the average standard of the three preceding performances was above the standard needed for a ‘good’ performance for the relevant station on 22 out of 74 (30%) occasions in the ‘previous high’ performances.

**FIGURE 2:**
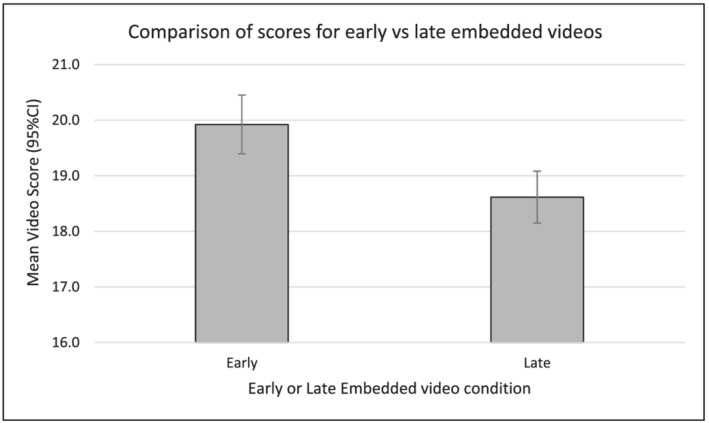
Comparison of scores for embedded videos in early and late conditions

GLM showed that average scores differed significantly between performances, ranging from 12.1 (95% CIs 8.7–15.5) to 27.5 (24.6–30.4), Wald *χ* ^2^ = 164.6 (*df* = 39), *p* < 0.001. Notably, the model‐estimated mean value for the highest scoring performance (i.e., 27.5) exceeded the scale maximum of 27 points, suggesting that ceiling effect may have curtailed scores for this performance. The mean estimated values for all other performances were <27, suggesting that this was unlikely to significantly bias the model. Average scores for examiners also differed significantly, ranging from 13.2 (10.1–16.4) to 26.1 (22.6–29.5), Wald *χ* ^2^ = 107.5 (29), *p* < 0.001. Scores differed significantly between performances early in the sequence (19.9 [19.4–20.4]) versus performances late in the sequence (18.6 [18.1–19.1]), Wald *χ* ^2^ = 12.5 (1), *p* < 0.001. This supported Hypothesis 3; examiners allocated lower scores for ‘late’ performances than for ‘early’ performances. This effect was small; Cohen's *d* = 1.3/3.7 = 0.35. To contextualise the magnitude of this difference, 361 out of 1520 (23.7%) of individual performances in the all within‐OSCE data were within a margin equal to or less than this difference (1.3 scale points) and could therefore potentially have their categorisation (pass/fail or fail/pass) altered by this magnitude of difference. These data are illustrated in Figure 2.

**FIGURE 3:**
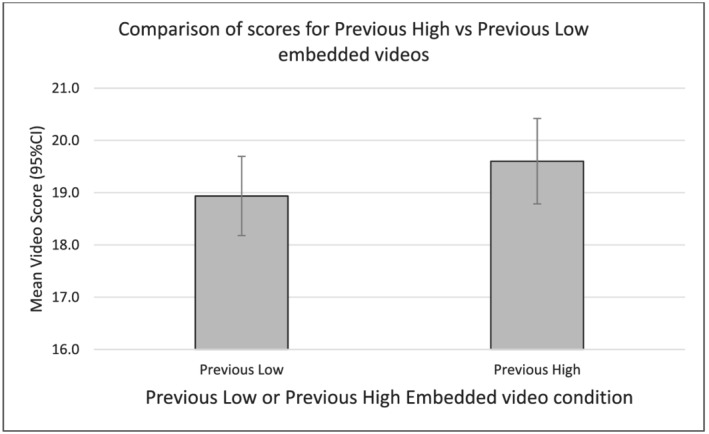
Comparison of scores for embedded videos under previous low and previous high conditions

Conversely, there was no significant difference in the scores given to video performances when they were preceded by high scoring performances (mean = 19.5 [95% CIs 18.7–20.4]) versus low scoring performance (18.9 [18.2–19.7], Wald *χ* ^2^ = 0.70 [1], *p* = 0.40). As a result, there was no evidence to support Hypothesis 4 or the presence of contrast effects. These data are illustrated in Figure 3. Re‐running the model including an interaction of the variables sequence × contrast showed an identical pattern of main effects. The interaction sequence × contrast was not significant (Wald *χ* ^2^ = 0.67 [1], *p* = 0.41). As a result, Hypothesis 5 was not supported.

## 4 DISCUSSION

### 4.1 Summary of findings

In this study we have used secondary data analysis to examine the presence of contrast and DRIFT effects within OSCE data, using both observational and controlled methods. Although linear relationships in all within‐OSCE data were not significant, controlled comparison of early and late performances showed that embedded video performances received lower scores when scored late in the sequence compared to early in the sequence. The size of this effect was small (Cohen's *d* = 0.35) Controlled comparisons of the scores given to embedded video performances did not support the presence of contrast effects in these data, and there was no evidence to support an interaction between DRIFT effects and contrast effects.

### 4.2 Theoretical implications of findings

This study has shown two somewhat contradictory findings: partial support for DRIFT effects and lack of support for contrast effects.

Although prior research has variously shown scores increasing^5^ and decreasing^16^ over the course of an OSCE, this study found a small difference (Cohen's *d* = 0.35) in the scores allocated to the same (controlled) performances when seen late rather than early in the sequence (1.3 points [6.2%]). The control afforded by this approach clarifies that (at least in this instance) this small effect appears to have been attributable to a change in examiner behaviour rather than an increase in students' performances or some other factor such as a change in simulated patient behaviour.

It is unclear why DRIFT effects were demonstrated for (controlled) embedded video performances but not demonstrated by relationships within (uncontrolled) within‐OSCE data. This could have occurred because the effect was too subtle to detect within more pronounced uncontrolled candidate‐to‐candidate variations. Conversely, it could be postulated that the unblinded switch between live and video performances might have unduly influenced examiners' scoring, and as such, the observation is a methodological artefact. Although this switch in modality could conceivably induce a Hawthorne effect,^26^ where performance increases due to awareness of observation (and therefore potentially making examiners' more attentive whilst score video performances), it is hard to see how this could produce a differential effect on scores over time. As a result, we suggest that the former explanation may be more likely, whilst noting that the inconsistent result makes the observation somewhat tentative.

If this assertion is accepted, then it is interesting to speculate what influences might have caused the small effect of examiner DRIFT on the embedded video performances. McLaughlin et al.^5^ speculated that examiner fatigue might cause the observed decline in scoring. Although examiner fatigue has previously been reported in OSCEs,^27^, ^28^ it is an issue that has received comparatively little attention despite the well‐described cognitive load that examiners experience.^29^ If fatigue does mediate this effect, we may expect to see a less dramatic effect when scoring criteria are optimised to reduce cognitive load or the examining task is simplified in other ways, for example, through station design.^30^, ^31^ Alternatively, examiners have described uncertainty in what score to allocate^4^, ^32^, ^33^ and can be reluctant to allocate failing scores when they are not certain.^34^, ^35^ Evolution in examiners' frames of reference^36^ over the course of the OSCE might alter their judgements or provide the confidence to score more negatively. Equally, DRIFT effects are clearly variable,^17^ and it may be that multiple effects interact at different time to produce different overall effects. Indeed, the muted (embedded) and null (all within‐OSCE data) effects we observed could have arisen due to the overlay of multiple DRIFT effects, some increasing and some decreasing scores over time. As these mechanisms are currently speculative, mechanistic work is required to understand these influences further.

The lack of support for contrast effects in these data contradicts the findings of the majority of prior research on this topic.^6^, ^13^, ^14^ Again, it is useful to consider potential reasons why they did not occur in these data. Prior data have found them to occur across various levels of learners from pre‐medical school selection through undergraduate medical school to postgraduate study. Consequently, the level of the learners seems an unlikely explanation for the null effect. Second, they have been supported in structured exam contexts as well as workplace‐based assessments. Consequently, it seems unlikely that the effect is simply attributable to the assessment context, although it remains possible that specific features of the exam context could have contributed to preventing the effect. Examiners in this study may have had a more developed sense of the level of the learners than in prior work, or the examiner information (details of the case, scoring criteria, performance guidance for examiners) could have been clearer than in prior work, either of which could have mitigated the effect. Although these explanations are appealing, the degree of observed examiner variability runs somewhat counter to these putative explanations. It could be that there are aspects of the organisation of the student rotation or specific elements of the assessment format that are responsible. This would require further study. In workplace based assessments, in addition to judging trainees' performances, assessors must also diagnose the clinical case and ensure that the patient receives safe and effective clinical care.^37^ Consequently WPBA may be expected to exert a higher cognitive load than OSCEs,^30^ which could potentially render them more susceptible to contrast effects. Lastly, it could be that the natural variation in the standard of students' performance in this study was insufficient to induce the effect. Our observation that the average standard of preceding performances was consistent with either failing or good performances in the ‘previous low’ and ‘previous high’ groups on a minority of occasions is consistent with this explanation. Categorising preceding performances on their absolute level (good or poor) rather than their relative level (better or worse) could also potentially have produced different findings. As explained in the methods, we chose this method as prior research suggests that contrast effects occur at all levels of performance but are greatest where the difference between successive students was large.^14^ If the null effect arose due to insufficient variation in students' ability, then we might conclude that contrast effect may only be a significant issue where candidates of very disparate ability are examined together. Although it is not possible to draw firm conclusions on any of these speculations, two points are salient: First, contrast effects may be less ubiquitous than the prior research had suggested, and second, these findings do not exclude the potential for them to occur in other OSCE situations. As a result, ongoing vigilance for their impact is needed.

### 4.3 Practical implications

Although emphasis on the formative role of OSCEs has justifiably increased,^38^ ensuring that OSCEs provide a fair measure of learners' ability remains critical to their justification.^39^ Consequently, any undue impact of these effects on assessment decisions in OSCEs could challenge the chain of their validity.^40^ Consideration of the importance of these effects is required. All candidates have both a first station and a last station within an OSCE rotation, and so one could postulate that DRIFT effects may be expected to exert an equal influence on candidates' overall scores in OSCEs, thereby negating their importance. Although this may be true in many circumstances, two assessment situations could still lead to them exerting a potentially important effect. First, many institutions use ‘conjunctive passing rules’,^41^, ^42^ where candidates fail the exam if they fail a certain proportion of stations. As DRIFT effects might result in additional station fails for some students, this could produce unwarranted failure for some candidates. If determined to be of sufficient importance in some instances, this effect could be mitigated by either adjusting students' station‐level scores or the station‐level pass mark^43^ based on sequence position of each performance. Alternatively, a station‐level standard error of measurement (SEM) could be used to allow for the influence. Second, many institutions run serial cycles of the OSCE through the course of an exam session, with different candidates in each cycle (see schematic for an example). DRIFT effects might disadvantage students in later cycles, potentially thereby influencing assessment decisions. This effect could be negated by varying students' cycle allocation over the course of an OSCE (i.e., Student A is in Cycle 1 on Day 1 and Cycle 3 on Day 2, etc.) or between successive exams over a programme. Importantly, the small (and inconsistently observed) magnitude of the effect we have found in this study may be considered insufficiently important to warrant alterations of this nature, given that other effects (such as the number of OSCE stations^44^) are known to have a greater influence on reliability of the test. As a result, whilst adding additional stations or testing time could theoretically worsen the DRIFT effect we observed, the added gains of additional stations on the OSCE's reliability seem likely to outweigh this effect. Nonetheless, as DRIFT effects appear to vary across contexts, it is important that they are monitored, and action considered to mitigate their influence if a substantial effect were to arise.

The absence of contrast effects in these data is to some degree reassuring as it is harder to conceive of a way of designing an OSCE to mitigate their effect given that they may arise from examiners judging performances in series. Moreover, as a borderline candidate might conceivably follow several highly capable candidates around a rotation, they could in theory be disadvantaged by contrast effects on the majority of stations, which could have a correspondingly important influence on their overall outcome. Consequently, further work is required to establish the prevalence of contrast effects in OSCEs and to understand the conditions under which they do and do not occur before general recommendations can be made.

### 4.4 Limitations

The main limitation of this study emanates from the secondary data upon which the analyses were based. These originated from a single OSCE in a single context and were originally collected for a different purpose. Although the findings may not implicitly generalise to other settings, we believe that the design and participant population were typical of many undergraduate OSCE settings. Investigation of contrast effects relied on natural variation in the standard of the preceding performances examiners judged, rather than deliberate manipulation. Although this was ecologically valid, it could have contributed to the null result we observed if there was insufficient variability in preceding performances. Although this produced an average difference in the standard of preceding performances of 11%–13% (see Section 3), a greater difference might have produced contrast effects. We cannot exclude the possibility that contrast effects did occur for some performances on some occasions.

Comparisons of the scores given to embedded video performances relied on an assumption that when examiners switched, unblinded, between judging live and video‐based performances, their judgements were unaffected by the change in modality. Although several studies have supported the equivalence of video‐based and live performance judgements in health professionals' education,^45^, ^46^, ^47^ the lack of blinding and switch in modality are both limitations of the method.

We only examined for the presence of contrast or DRIFT effects in overall scores; we cannot exclude the possibility that contrast or DRIFT effects might have occurred within individual domains of the assessment and could therefore bias the scores within these domains. The importance of any such effect (were it to occur) would depend on the particular usage of domains scores within a given assessment. Additionally, we were not able to model the potential for overlaid positive and negative DRIFT effects. We cannot exclude the potential that the small/null DRIFT effects we have reported masked more pronounced effects in subsets of data.

### 4.5 Future research

Given the uncertainty around when, why and how both contrast and DRIFT effects may occur in assessments, future work should seek to more thoroughly establish the prevalence of these effects in OSCE exams and seek to determine conditions which mediate their presence and/or direction. Depending on the importance of effects that occur, further work might explore the cost–benefit relationship of measures to mitigate their effects in practice. As several institutions have recently explored the potential for online OSCEs, these may offer an opportunity to replicate the study, whilst blinding examiners to the presence of comparison performances (as these could potentially be ‘hidden’ amongst other on‐screen performances). This could overcome one of the limitations mentioned above. Further work could explore the presence or absence of domain‐level effects.

### 4.6 Conclusions

Our findings suggest that the DRIFT we observed has a small influence on students' OSCE scores that can be causally attributed to examiner. Although the magnitude of DRIFT effects will not always have important consequences, they can reduce the precision of scores and have the potential to produce an unfair influence that should be considered within quality assurance of OSCEs.

Conversely, although contrast effects may importantly bias examiners' scores in some instances, they appear less ubiquitous than previously suggested. Consequently, more research is required to determine the prevalence and mediators of both influences so that assessment design can be used to avoid or limit the impact of their occurrence or so that mitigating interventions can be developed.

As both effects appear to be contextually variable, their presence or absence should be monitored as part of quality assurance processes to ensure the fairness and validity of assessment outcomes in OSCEs.

## CONFLICT OF INTERESTS

The authors declare no conflicts of interest.

## ETHICS STATEMENT

This study did not recruit any new human participants. Ethical approval for this analysis was granted within the approval for the original study (Keele ERP 2413). Within the original study, participation in filming (by students, examiners and simulated patients) was voluntary and participants provided consent and had the right to withdraw. Video scoring by examiners was also voluntary, and they also provided consent and could withdraw. Data were pseudonymised before analysis, and all identifiable data were treated confidentially and stored securely.

## AUTHOR CONTRIBUTIONS

PY substantially contributed to the conception, analysis, interpretation, drafting and critical revision of the paper. He has given final approval to the manuscript and agrees to be accountable for the work.

AM substantially contributed to the conception and interpretation and critical revisions of the paper. She has given final approval to the manuscript and agrees to be accountable for the work.

NC substantially contributed to the conception, interpretation and critical revision of the paper. She has given final approval to the manuscript and agrees to be accountable for the work.

GM substantially contributed to the conception, analysis, interpretation, drafting and critical revision of the paper. He has given final approval to the manuscript and agrees to be accountable for the work.

RF substantially contributed to the conception, interpretation and critical revision of the paper. He has given final approval to the manuscript and agrees to be accountable for the work.

RM substantially contributed to the conception, analysis, interpretation, drafting and critical revision of the paper. He has given final approval to the manuscript and agrees to be accountable for the work.
